# Species conservation profile of the rare and endemic trapdoor spider *Calathotarsus simoni* (Araneae, Migidae) from Central Argentina

**DOI:** 10.3897/BDJ.5.e14790

**Published:** 2017-10-24

**Authors:** Nelson Ferretti, Gabriel Pompozzi, Pedro Cardoso

**Affiliations:** 1 Centro de Estudios Parasitológicos y de Vectores CEPAVE, La Plata, Argentina; 2 IUCN SSC Spider & Scorpion Specialist Group, Helsinki, Finland; 3 Instituto de Ciencias Biológicas y Biomédicas del Sur, Bahia Blanca, Argentina; 4 Finnish Museum of Natural History, University of Helsinki,, Helsinki, Finland

**Keywords:** South America, IUCN, grassland, red list, mygalomorph spider

## Abstract

**Background:**

*Calathotarsus simoni* Schiapelli & Gerschman 1975 is the only species of Migidae in Argentina. It is a rare and endemic spider only found in relict grassland of mountain systems in the province of Buenos Aires. This species is a habitat specialist spider occupying specific areas with certain microclimatic conditions on hilly and rocky grassland areas at about 500-1500 meters above sea level.

**New information:**

The observed extent of occurrence (EOO) is 7207 km^2^ and the area of occupancy (AOO) is 16 km^2^. Two locations are identified based on the threat event related to the invasion of the species habitat by alien woody plants. In addition, intensive cattle production and agriculture also constitute relevant threats to the species.

## Introduction

Trapdoor spiders belong to the infraorder Mygalomorphae and include species that dig burrows into the ground, sealed with a lid or “trapdoor”. The spiders live in these burrows and can emerge from them to feed. They are long-lived, slow reproducing, burrowing spiders with several species of conservation concern in different parts of the world (Harrison et al. 2017). Members of the mygalomorph family Migidae are known from Australia, Africa, Madagascar, New Zealand, New Caledonia and the southern cone of South America: almost all parts of the former supercontinent Gondwanaland, except the Indian subcontinent and Antarctica (Griswold and Ledford 2001). *Calathotarsus* (Migidae) are medium-sized spiders (12–20 mm) with an arched caput in the female, wide ocular area, and with rows of setae on the caput (Schiapelli and Gerschman de Pikelín 1973). *Calathotarsus simoni* is an endemic species from central Argentina living in just some specific areas with certain microclimatic conditions found at hilly and rocky grassland areas at about 500-1500 meters above sea level (Ferretti et al. 2014a). The hilly environments where this species occupy are suffering many threats that could led to future population decreases.

## Species Conservation Profiles

### Calathotarsus simoni

#### Species information

Scientific name: Calathotarsus simoni

Species authority: Schiapelli & Gerschman de Pikelín, 1975

Common names: "Araña albañil", trapdoor spider

Kingdom: Animalia

Phylum: Arthropoda

Class: Arachnida

Order: Araneae

Family: Migidae

**Figure 1.**
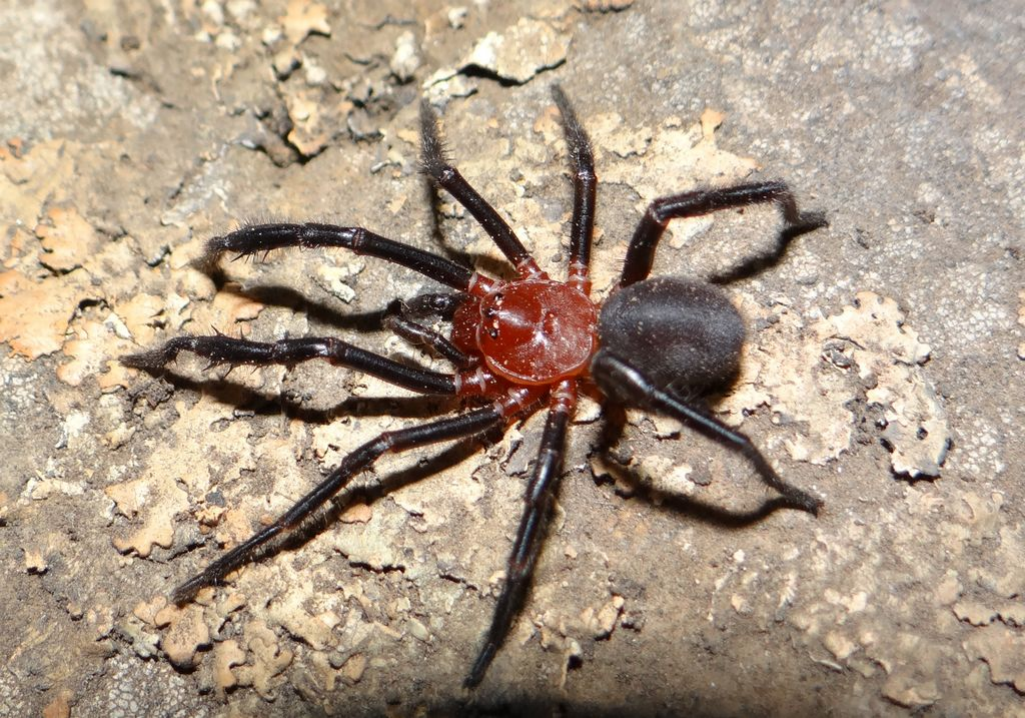
Adult male of *Calathotarsus simoni*

Taxonomic notes: This species has not been under taxonomical issues or changes since its original description. It was described in 1975 based on females and one male from Sierra de la Ventana, Tornquist, Buenos Aires, Argentina. It is the only species of the family present in Argentina. It is a medium-sized spider (12–20 mm) with an arched caput in the female, wide ocular area, and with rows of setae on the caput (Schiapelli and Gerschman de Pikelín 1973) Figs 1, 2. The thoracic fovea is simple or may have a weak posterior extension. The cheliceral fang furrow has denticles between the tooth rows and intercheliceral tumescence in the male, the cuspules of the pedipalp coxae are clustered near the base (Griswold and Ledford 2001). Males of this species have a conspicuous coloration with a red-orange carapace, chelicerae, coxae and trochanters Fig. 1.

Region for assessment: Global

#### Editor & Reviewers

##### Reviewers

Reviewers: Dr. Manju Siliwal, email: manjusiliwal@gmail.comDr. Marshal Hedin, email: mhedin@mail.sdsu.edu

##### Editor

Editor: Jeremy Miller

#### Reviewers

Reviewers: Dr. Manju Siliwal, email: manjusiliwal@gmail.comDr. Marshal Hedin, email: mhedin@mail.sdsu.edu

#### Editor

Editor: Jeremy Miller

#### Geographic range

Biogeographic realm: Neotropical

Countries: Argentina

**Figure 3.**
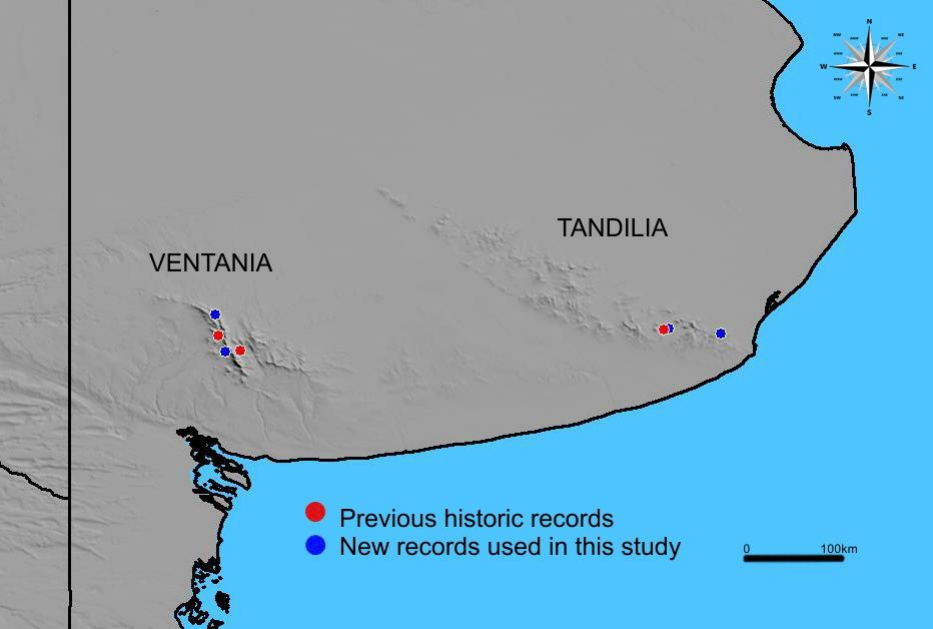
Known distribution of *C. simoni*

Map of records (image): Fig. 3

Map of records (Google Earth): Suppl. material 1

Basis of EOO and AOO: Observed

Basis (narrative): This species endemic to southern Buenos Aires province, Argentina, is only found in grassland of mountainous systems in this region. The species was originally described for two sites (Cerro Negro and Fortín Chaco) from Tornquist locality at the Ventania mountain system and two sites (La Barrosa and Sierra de la Cruz) from Balcarce locality at the Tandilia mountain system. These records correspond to the original publication of Schiapelli and Gerschman de Pikelín, 1975. From that historic records, recent field campaings allowed to us to record specimens from only one site at Tandilia mountanious system (La Barrosa). Additionally, the species was also found in three new sites: Funke and Hinojo (Ventania) and Paititi (Tandilia), all of them located near original collection sites. Despite our extensive study of *C. simoni* specimens (those from Ferretti et al. 2014a) together with the examination of material stored in Museums (MACN, Museo Argentino de Ciencias Naturales, Buenos Aires, Argentina and LZI, Laboratorio de Zoología de Invertebrados II, Bahía Blanca, Buenos Aires, Argentina) and original records (Ferretti in prep.), we have not been able to find additional records of *C. simoni* outside these small areas. Given the species rarity and the extensive studies (Ferretti et al. 2012, Ferretti et al. 2014a, Ferretti et al. 2014b) carried out to identify its geographical range, we used the new observed and confirmed occurrence records (Funke, Hinojo, La Barrosa and Paititi) to calculate both the extent of occurrence (EOO) and the area of occupancy (AOO), using a 2x2 km grid, as implemented in the *red* R package (Cardoso 2017).

Range description: *Calathotarsus simoni* is endemic to the province of Buenos Aires, Argentina, limited to grasslands of mountain systems. The species is recorded in two nearby localities in the mountain system of Ventania and two other in the system of Tandilia.

#### New occurrences

##### Material

Type status: Other material

###### Occurrence

catalogNumber: LZI283

recordedBy: Pompozzi

individualCount: 2

sex: female

lifeStage: adult

###### Taxon

scientificNameID: Calathotarsus simoni

kingdom: Animalia

phylum: Arthropoda

class: Arachnida

order: Araneae

family: Migidae

genus: Calathotarsus

specificEpithet: simoni

scientificNameAuthorship: Schiapelli and Gerschman de Pikelín, 197

###### Location

continent: South America

country: Argentina

stateProvince: Buenos Aires

county: Tornquist

municipality: Tornquist

locality: Funke Ranch

verbatimCoordinates: 38°4'20.12"S 62°3'11.07"W

verbatimSRS: WGS84

decimalLatitude: -37.927744444444

decimalLongitude: -61.946925

georeferenceProtocol: GPS

###### Identification

identifiedBy: Nelson Ferretti

dateIdentified: 2012

###### Geological context

###### Event

samplingProtocol: Hand collected

eventDate: 2012-05-03

year: 2012

month: 5

day: 3

###### Record Level

collectionCode: LZI

##### Occurrence

catalogNumber: LZI283

recordedBy: Pompozzi

individualCount: 2

sex: female

lifeStage: adult

##### Taxon

scientificNameID: Calathotarsus simoni

kingdom: Animalia

phylum: Arthropoda

class: Arachnida

order: Araneae

family: Migidae

genus: Calathotarsus

specificEpithet: simoni

scientificNameAuthorship: Schiapelli and Gerschman de Pikelín, 197

##### Location

continent: South America

country: Argentina

stateProvince: Buenos Aires

county: Tornquist

municipality: Tornquist

locality: Funke Ranch

verbatimCoordinates: 38°4'20.12"S 62°3'11.07"W

verbatimSRS: WGS84

decimalLatitude: -37.927744444444

decimalLongitude: -61.946925

georeferenceProtocol: GPS

##### Identification

identifiedBy: Nelson Ferretti

dateIdentified: 2012

##### Geological context

##### Event

samplingProtocol: Hand collected

eventDate: 2012-05-03

year: 2012

month: 5

day: 3

##### Record Level

collectionCode: LZI

##### Material

Type status: Other material

###### Occurrence

catalogNumber: LZI315

recordedBy: Copperi

individualCount: 1

sex: female

lifeStage: adult

###### Taxon

scientificNameID: Calathotarsus simoni

kingdom: Animalia

phylum: Arthropoda

class: Arachnida

order: Araneae

family: Migidae

genus: Calathotarsus

specificEpithet: simoni

scientificNameAuthorship: Schiapelli and Gerschman de Pikelín, 197

###### Location

continent: South America

country: Argentina

stateProvince: Buenos Aires

county: Saavedra

municipality: Saavedra

locality: Abra del Hinojo

verbatimCoordinates: 37°45'34.16"S, 62°8'27.16"W

verbatimSRS: WGS84

decimalLatitude: -36.240511111111

decimalLongitude: -61.859122222222

georeferenceProtocol: GPS

###### Identification

identifiedBy: Nelson Ferretti

dateIdentified: 2014

###### Geological context

###### Event

samplingProtocol: Hand collected

eventDate: 2014-02-27

year: 2014

month: 2

day: 27

###### Record Level

collectionCode: LZI

##### Occurrence

catalogNumber: LZI315

recordedBy: Copperi

individualCount: 1

sex: female

lifeStage: adult

##### Taxon

scientificNameID: Calathotarsus simoni

kingdom: Animalia

phylum: Arthropoda

class: Arachnida

order: Araneae

family: Migidae

genus: Calathotarsus

specificEpithet: simoni

scientificNameAuthorship: Schiapelli and Gerschman de Pikelín, 197

##### Location

continent: South America

country: Argentina

stateProvince: Buenos Aires

county: Saavedra

municipality: Saavedra

locality: Abra del Hinojo

verbatimCoordinates: 37°45'34.16"S, 62°8'27.16"W

verbatimSRS: WGS84

decimalLatitude: -36.240511111111

decimalLongitude: -61.859122222222

georeferenceProtocol: GPS

##### Identification

identifiedBy: Nelson Ferretti

dateIdentified: 2014

##### Geological context

##### Event

samplingProtocol: Hand collected

eventDate: 2014-02-27

year: 2014

month: 2

day: 27

##### Record Level

collectionCode: LZI

##### Material

Type status: Other material

###### Occurrence

catalogNumber: LZI383

recordedBy: Peralta

individualCount: 1

sex: male

lifeStage: adult

###### Taxon

scientificNameID: Calathotarsus simoni

kingdom: Animalia

phylum: Arthropoda

class: Arachnida

order: Araneae

family: Migidae

genus: Calathotarsus

specificEpithet: simoni

scientificNameAuthorship: Schiapelli and Gerschman de Pikelín, 197

###### Location

continent: South America

country: Argentina

stateProvince: Buenos Aires

county: General Pueyrredón

municipality: Sierra de los Padres

locality: Paititi

verbatimCoordinates: 37°55'11.55"S 57°49'21.55"W?

verbatimSRS: WGS84

decimalLatitude: -36.080125

decimalLongitude: -56.177347222222

georeferenceProtocol: GPS

###### Identification

identifiedBy: Nelson Ferretti

dateIdentified: 2015

###### Geological context

###### Event

samplingProtocol: Pitfall trap

eventDate: 2015-06-16

year: 2015

month: 6

day: 16

###### Record Level

collectionCode: LZI

##### Occurrence

catalogNumber: LZI383

recordedBy: Peralta

individualCount: 1

sex: male

lifeStage: adult

##### Taxon

scientificNameID: Calathotarsus simoni

kingdom: Animalia

phylum: Arthropoda

class: Arachnida

order: Araneae

family: Migidae

genus: Calathotarsus

specificEpithet: simoni

scientificNameAuthorship: Schiapelli and Gerschman de Pikelín, 197

##### Location

continent: South America

country: Argentina

stateProvince: Buenos Aires

county: General Pueyrredón

municipality: Sierra de los Padres

locality: Paititi

verbatimCoordinates: 37°55'11.55"S 57°49'21.55"W?

verbatimSRS: WGS84

decimalLatitude: -36.080125

decimalLongitude: -56.177347222222

georeferenceProtocol: GPS

##### Identification

identifiedBy: Nelson Ferretti

dateIdentified: 2015

##### Geological context

##### Event

samplingProtocol: Pitfall trap

eventDate: 2015-06-16

year: 2015

month: 6

day: 16

##### Record Level

collectionCode: LZI

##### Material

Type status: Other material

###### Occurrence

catalogNumber: MACN6696

recordedBy: Cesari, Dominguez

individualCount: 1

sex: male

lifeStage: adult

###### Taxon

scientificNameID: Calathotarsus simoni

kingdom: Animalia

phylum: Arthropoda

class: Arachnida

order: Araneae

family: Migidae

genus: Calathotarsus

specificEpithet: simoni

scientificNameAuthorship: Schiapelli and Gerschman de Pikelín, 198

###### Location

continent: South America

country: Argentina

stateProvince: Buenos Aires

county: Tornquist

municipality: Tornquist

locality: Cerro Negro

###### Identification

###### Geological context

###### Event

eventDate: 1974-04

year: 1974

month: 4

###### Record Level

collectionCode: MACN

##### Occurrence

catalogNumber: MACN6696

recordedBy: Cesari, Dominguez

individualCount: 1

sex: male

lifeStage: adult

##### Taxon

scientificNameID: Calathotarsus simoni

kingdom: Animalia

phylum: Arthropoda

class: Arachnida

order: Araneae

family: Migidae

genus: Calathotarsus

specificEpithet: simoni

scientificNameAuthorship: Schiapelli and Gerschman de Pikelín, 198

##### Location

continent: South America

country: Argentina

stateProvince: Buenos Aires

county: Tornquist

municipality: Tornquist

locality: Cerro Negro

##### Identification

##### Geological context

##### Event

eventDate: 1974-04

year: 1974

month: 4

##### Record Level

collectionCode: MACN

##### Material

Type status: Other material

###### Occurrence

catalogNumber: MACN36114

recordedBy: Cesari

individualCount: 3

sex: 1 male, 2 females

lifeStage: adult

###### Taxon

scientificNameID: Calathotarsus simoni

kingdom: Animalia

phylum: Arthropoda

class: Arachnida

order: Araneae

family: Migidae

genus: Calathotarsus

specificEpithet: simoni

scientificNameAuthorship: Schiapelli and Gerschman de Pikelín, 199

###### Location

continent: South America

country: Argentina

stateProvince: Buenos Aires

county: Tornquist

municipality: Tornquist

locality: Cerro Negro

###### Identification

identifiedBy: Pablo Goloboff

###### Geological context

###### Event

eventDate: 1974-04

year: 1974

month: 4

###### Record Level

collectionCode: MACN

##### Occurrence

catalogNumber: MACN36114

recordedBy: Cesari

individualCount: 3

sex: 1 male, 2 females

lifeStage: adult

##### Taxon

scientificNameID: Calathotarsus simoni

kingdom: Animalia

phylum: Arthropoda

class: Arachnida

order: Araneae

family: Migidae

genus: Calathotarsus

specificEpithet: simoni

scientificNameAuthorship: Schiapelli and Gerschman de Pikelín, 199

##### Location

continent: South America

country: Argentina

stateProvince: Buenos Aires

county: Tornquist

municipality: Tornquist

locality: Cerro Negro

##### Identification

identifiedBy: Pablo Goloboff

##### Geological context

##### Event

eventDate: 1974-04

year: 1974

month: 4

##### Record Level

collectionCode: MACN

##### Material

Type status: Other material

###### Occurrence

catalogNumber: MACN36113

recordedBy: Cesari

individualCount: 1

sex: male

lifeStage: adult

###### Taxon

scientificNameID: Calathotarsus simoni

kingdom: Animalia

phylum: Arthropoda

class: Arachnida

order: Araneae

family: Migidae

genus: Calathotarsus

specificEpithet: simoni

scientificNameAuthorship: Schiapelli and Gerschman de Pikelín, 200

###### Location

continent: South America

country: Argentina

stateProvince: Buenos Aires

county: Tornquist

municipality: Tornquist

locality: Cerro Negro

###### Identification

###### Geological context

###### Event

eventDate: 1974-04

year: 1974

month: 4

###### Record Level

collectionCode: MACN

##### Occurrence

catalogNumber: MACN36113

recordedBy: Cesari

individualCount: 1

sex: male

lifeStage: adult

##### Taxon

scientificNameID: Calathotarsus simoni

kingdom: Animalia

phylum: Arthropoda

class: Arachnida

order: Araneae

family: Migidae

genus: Calathotarsus

specificEpithet: simoni

scientificNameAuthorship: Schiapelli and Gerschman de Pikelín, 200

##### Location

continent: South America

country: Argentina

stateProvince: Buenos Aires

county: Tornquist

municipality: Tornquist

locality: Cerro Negro

##### Identification

##### Geological context

##### Event

eventDate: 1974-04

year: 1974

month: 4

##### Record Level

collectionCode: MACN

##### Material

Type status: Other material

###### Occurrence

catalogNumber: MACN6698

recordedBy: Maury

individualCount: 2

sex: female

lifeStage: adult

###### Taxon

scientificNameID: Calathotarsus simoni

kingdom: Animalia

phylum: Arthropoda

class: Arachnida

order: Araneae

family: Migidae

genus: Calathotarsus

specificEpithet: simoni

scientificNameAuthorship: Schiapelli and Gerschman de Pikelín, 201

###### Location

continent: South America

country: Argentina

stateProvince: Buenos Aires

county: Tornquist

municipality: Tornquist

locality: Fortin Chaco

###### Identification

###### Geological context

###### Event

eventDate: 1973-03

year: 1973

month: 3

###### Record Level

collectionCode: MACN

##### Occurrence

catalogNumber: MACN6698

recordedBy: Maury

individualCount: 2

sex: female

lifeStage: adult

##### Taxon

scientificNameID: Calathotarsus simoni

kingdom: Animalia

phylum: Arthropoda

class: Arachnida

order: Araneae

family: Migidae

genus: Calathotarsus

specificEpithet: simoni

scientificNameAuthorship: Schiapelli and Gerschman de Pikelín, 201

##### Location

continent: South America

country: Argentina

stateProvince: Buenos Aires

county: Tornquist

municipality: Tornquist

locality: Fortin Chaco

##### Identification

##### Geological context

##### Event

eventDate: 1973-03

year: 1973

month: 3

##### Record Level

collectionCode: MACN

##### Material

Type status: Other material

###### Occurrence

catalogNumber: MACN6643

recordedBy: Maury, Cesari

individualCount: 1

sex: female

lifeStage: adult

###### Taxon

scientificNameID: Calathotarsus simoni

kingdom: Animalia

phylum: Arthropoda

class: Arachnida

order: Araneae

family: Migidae

genus: Calathotarsus

specificEpithet: simoni

scientificNameAuthorship: Schiapelli and Gerschman de Pikelín, 202

###### Location

continent: South America

country: Argentina

stateProvince: Buenos Aires

county: Tornquist

municipality: Tornquist

locality: Fortin Chaco

###### Identification

###### Geological context

###### Event

eventDate: 1974-04

year: 1974

month: 4

###### Record Level

collectionCode: MACN

##### Occurrence

catalogNumber: MACN6643

recordedBy: Maury, Cesari

individualCount: 1

sex: female

lifeStage: adult

##### Taxon

scientificNameID: Calathotarsus simoni

kingdom: Animalia

phylum: Arthropoda

class: Arachnida

order: Araneae

family: Migidae

genus: Calathotarsus

specificEpithet: simoni

scientificNameAuthorship: Schiapelli and Gerschman de Pikelín, 202

##### Location

continent: South America

country: Argentina

stateProvince: Buenos Aires

county: Tornquist

municipality: Tornquist

locality: Fortin Chaco

##### Identification

##### Geological context

##### Event

eventDate: 1974-04

year: 1974

month: 4

##### Record Level

collectionCode: MACN

##### Material

Type status: Other material

###### Occurrence

catalogNumber: MACN6617

recordedBy: Cesari

individualCount: 1

sex: female

lifeStage: adult

###### Taxon

scientificNameID: Calathotarsus simoni

kingdom: Animalia

phylum: Arthropoda

class: Arachnida

order: Araneae

family: Migidae

genus: Calathotarsus

specificEpithet: simoni

scientificNameAuthorship: Schiapelli and Gerschman de Pikelín, 203

###### Location

continent: South America

country: Argentina

stateProvince: Buenos Aires

county: Balcarce

municipality: Balcarce

locality: Sierra de la Cruz

###### Identification

###### Geological context

###### Event

eventDate: 1972-02

year: 1972

month: 2

###### Record Level

collectionCode: MACN

##### Occurrence

catalogNumber: MACN6617

recordedBy: Cesari

individualCount: 1

sex: female

lifeStage: adult

##### Taxon

scientificNameID: Calathotarsus simoni

kingdom: Animalia

phylum: Arthropoda

class: Arachnida

order: Araneae

family: Migidae

genus: Calathotarsus

specificEpithet: simoni

scientificNameAuthorship: Schiapelli and Gerschman de Pikelín, 203

##### Location

continent: South America

country: Argentina

stateProvince: Buenos Aires

county: Balcarce

municipality: Balcarce

locality: Sierra de la Cruz

##### Identification

##### Geological context

##### Event

eventDate: 1972-02

year: 1972

month: 2

##### Record Level

collectionCode: MACN

##### Material

Type status: Other material

###### Occurrence

catalogNumber: MACN6622

recordedBy: Maury, Cesari, Dominguez

individualCount: 1

sex: female

lifeStage: adult

###### Taxon

scientificNameID: Calathotarsus simoni

kingdom: Animalia

phylum: Arthropoda

class: Arachnida

order: Araneae

family: Migidae

genus: Calathotarsus

specificEpithet: simoni

scientificNameAuthorship: Schiapelli and Gerschman de Pikelín, 204

###### Location

continent: South America

country: Argentina

stateProvince: Buenos Aires

county: Tornquist

municipality: Tornquist

locality: Fortin Chaco

###### Identification

###### Geological context

###### Event

###### Record Level

collectionCode: MACN

##### Occurrence

catalogNumber: MACN6622

recordedBy: Maury, Cesari, Dominguez

individualCount: 1

sex: female

lifeStage: adult

##### Taxon

scientificNameID: Calathotarsus simoni

kingdom: Animalia

phylum: Arthropoda

class: Arachnida

order: Araneae

family: Migidae

genus: Calathotarsus

specificEpithet: simoni

scientificNameAuthorship: Schiapelli and Gerschman de Pikelín, 204

##### Location

continent: South America

country: Argentina

stateProvince: Buenos Aires

county: Tornquist

municipality: Tornquist

locality: Fortin Chaco

##### Identification

##### Geological context

##### Event

##### Record Level

collectionCode: MACN

##### Material

Type status: Other material

###### Occurrence

catalogNumber: MACN6697

recordedBy: Maury, Cesari, Dominguez

individualCount: 2

sex: female

lifeStage: adult

###### Taxon

scientificNameID: Calathotarsus simoni

kingdom: Animalia

phylum: Arthropoda

class: Arachnida

order: Araneae

family: Migidae

genus: Calathotarsus

specificEpithet: simoni

scientificNameAuthorship: Schiapelli and Gerschman de Pikelín, 205

###### Location

continent: South America

country: Argentina

stateProvince: Buenos Aires

county: Balcarce

municipality: Balcarce

locality: La Barrosa

###### Identification

###### Geological context

###### Event

eventDate: 1974-07

year: 1974

month: 7

###### Record Level

collectionCode: MACN

##### Occurrence

catalogNumber: MACN6697

recordedBy: Maury, Cesari, Dominguez

individualCount: 2

sex: female

lifeStage: adult

##### Taxon

scientificNameID: Calathotarsus simoni

kingdom: Animalia

phylum: Arthropoda

class: Arachnida

order: Araneae

family: Migidae

genus: Calathotarsus

specificEpithet: simoni

scientificNameAuthorship: Schiapelli and Gerschman de Pikelín, 205

##### Location

continent: South America

country: Argentina

stateProvince: Buenos Aires

county: Balcarce

municipality: Balcarce

locality: La Barrosa

##### Identification

##### Geological context

##### Event

eventDate: 1974-07

year: 1974

month: 7

##### Record Level

collectionCode: MACN

##### Material

Type status: Other material

###### Occurrence

catalogNumber: LZI543

recordedBy: Ferretti

individualCount: 1

sex: female

lifeStage: adult

###### Taxon

scientificNameID: Calathotarsus simoni

kingdom: Animalia

phylum: Arthropoda

class: Arachnida

order: Araneae

family: Migidae

genus: Calathotarsus

specificEpithet: simoni

scientificNameAuthorship: Schiapelli and Gerschman de Pikelín, 206

###### Location

continent: South America

country: Argentina

stateProvince: Buenos Aires

county: Balcarce

municipality: Balcarce

locality: La Barrosa

verbatimCoordinates: 37°52'37"S 58°15'40"W?

verbatimSRS: WGS84

decimalLatitude: -36.123055555556

decimalLongitude: -57.738888888889

georeferenceProtocol: GPS

###### Identification

identifiedBy: Ferretti

dateIdentified: 2015

###### Geological context

###### Event

samplingProtocol: Hand collected

eventDate: 2015-11-12

year: 2015

month: 11

day: 12

###### Record Level

collectionCode: LZI

##### Occurrence

catalogNumber: LZI543

recordedBy: Ferretti

individualCount: 1

sex: female

lifeStage: adult

##### Taxon

scientificNameID: Calathotarsus simoni

kingdom: Animalia

phylum: Arthropoda

class: Arachnida

order: Araneae

family: Migidae

genus: Calathotarsus

specificEpithet: simoni

scientificNameAuthorship: Schiapelli and Gerschman de Pikelín, 206

##### Location

continent: South America

country: Argentina

stateProvince: Buenos Aires

county: Balcarce

municipality: Balcarce

locality: La Barrosa

verbatimCoordinates: 37°52'37"S 58°15'40"W?

verbatimSRS: WGS84

decimalLatitude: -36.123055555556

decimalLongitude: -57.738888888889

georeferenceProtocol: GPS

##### Identification

identifiedBy: Ferretti

dateIdentified: 2015

##### Geological context

##### Event

samplingProtocol: Hand collected

eventDate: 2015-11-12

year: 2015

month: 11

day: 12

##### Record Level

collectionCode: LZI

#### Material

Type status: Other material

##### Occurrence

catalogNumber: LZI283

recordedBy: Pompozzi

individualCount: 2

sex: female

lifeStage: adult

##### Taxon

scientificNameID: Calathotarsus simoni

kingdom: Animalia

phylum: Arthropoda

class: Arachnida

order: Araneae

family: Migidae

genus: Calathotarsus

specificEpithet: simoni

scientificNameAuthorship: Schiapelli and Gerschman de Pikelín, 197

##### Location

continent: South America

country: Argentina

stateProvince: Buenos Aires

county: Tornquist

municipality: Tornquist

locality: Funke Ranch

verbatimCoordinates: 38°4'20.12"S 62°3'11.07"W

verbatimSRS: WGS84

decimalLatitude: -37.927744444444

decimalLongitude: -61.946925

georeferenceProtocol: GPS

##### Identification

identifiedBy: Nelson Ferretti

dateIdentified: 2012

##### Geological context

##### Event

samplingProtocol: Hand collected

eventDate: 2012-05-03

year: 2012

month: 5

day: 3

##### Record Level

collectionCode: LZI

#### Occurrence

catalogNumber: LZI283

recordedBy: Pompozzi

individualCount: 2

sex: female

lifeStage: adult

#### Taxon

scientificNameID: Calathotarsus simoni

kingdom: Animalia

phylum: Arthropoda

class: Arachnida

order: Araneae

family: Migidae

genus: Calathotarsus

specificEpithet: simoni

scientificNameAuthorship: Schiapelli and Gerschman de Pikelín, 197

#### Location

continent: South America

country: Argentina

stateProvince: Buenos Aires

county: Tornquist

municipality: Tornquist

locality: Funke Ranch

verbatimCoordinates: 38°4'20.12"S 62°3'11.07"W

verbatimSRS: WGS84

decimalLatitude: -37.927744444444

decimalLongitude: -61.946925

georeferenceProtocol: GPS

#### Identification

identifiedBy: Nelson Ferretti

dateIdentified: 2012

#### Geological context

#### Event

samplingProtocol: Hand collected

eventDate: 2012-05-03

year: 2012

month: 5

day: 3

#### Record Level

collectionCode: LZI

#### Material

Type status: Other material

##### Occurrence

catalogNumber: LZI315

recordedBy: Copperi

individualCount: 1

sex: female

lifeStage: adult

##### Taxon

scientificNameID: Calathotarsus simoni

kingdom: Animalia

phylum: Arthropoda

class: Arachnida

order: Araneae

family: Migidae

genus: Calathotarsus

specificEpithet: simoni

scientificNameAuthorship: Schiapelli and Gerschman de Pikelín, 197

##### Location

continent: South America

country: Argentina

stateProvince: Buenos Aires

county: Saavedra

municipality: Saavedra

locality: Abra del Hinojo

verbatimCoordinates: 37°45'34.16"S, 62°8'27.16"W

verbatimSRS: WGS84

decimalLatitude: -36.240511111111

decimalLongitude: -61.859122222222

georeferenceProtocol: GPS

##### Identification

identifiedBy: Nelson Ferretti

dateIdentified: 2014

##### Geological context

##### Event

samplingProtocol: Hand collected

eventDate: 2014-02-27

year: 2014

month: 2

day: 27

##### Record Level

collectionCode: LZI

#### Occurrence

catalogNumber: LZI315

recordedBy: Copperi

individualCount: 1

sex: female

lifeStage: adult

#### Taxon

scientificNameID: Calathotarsus simoni

kingdom: Animalia

phylum: Arthropoda

class: Arachnida

order: Araneae

family: Migidae

genus: Calathotarsus

specificEpithet: simoni

scientificNameAuthorship: Schiapelli and Gerschman de Pikelín, 197

#### Location

continent: South America

country: Argentina

stateProvince: Buenos Aires

county: Saavedra

municipality: Saavedra

locality: Abra del Hinojo

verbatimCoordinates: 37°45'34.16"S, 62°8'27.16"W

verbatimSRS: WGS84

decimalLatitude: -36.240511111111

decimalLongitude: -61.859122222222

georeferenceProtocol: GPS

#### Identification

identifiedBy: Nelson Ferretti

dateIdentified: 2014

#### Geological context

#### Event

samplingProtocol: Hand collected

eventDate: 2014-02-27

year: 2014

month: 2

day: 27

#### Record Level

collectionCode: LZI

#### Material

Type status: Other material

##### Occurrence

catalogNumber: LZI383

recordedBy: Peralta

individualCount: 1

sex: male

lifeStage: adult

##### Taxon

scientificNameID: Calathotarsus simoni

kingdom: Animalia

phylum: Arthropoda

class: Arachnida

order: Araneae

family: Migidae

genus: Calathotarsus

specificEpithet: simoni

scientificNameAuthorship: Schiapelli and Gerschman de Pikelín, 197

##### Location

continent: South America

country: Argentina

stateProvince: Buenos Aires

county: General Pueyrredón

municipality: Sierra de los Padres

locality: Paititi

verbatimCoordinates: 37°55'11.55"S 57°49'21.55"W?

verbatimSRS: WGS84

decimalLatitude: -36.080125

decimalLongitude: -56.177347222222

georeferenceProtocol: GPS

##### Identification

identifiedBy: Nelson Ferretti

dateIdentified: 2015

##### Geological context

##### Event

samplingProtocol: Pitfall trap

eventDate: 2015-06-16

year: 2015

month: 6

day: 16

##### Record Level

collectionCode: LZI

#### Occurrence

catalogNumber: LZI383

recordedBy: Peralta

individualCount: 1

sex: male

lifeStage: adult

#### Taxon

scientificNameID: Calathotarsus simoni

kingdom: Animalia

phylum: Arthropoda

class: Arachnida

order: Araneae

family: Migidae

genus: Calathotarsus

specificEpithet: simoni

scientificNameAuthorship: Schiapelli and Gerschman de Pikelín, 197

#### Location

continent: South America

country: Argentina

stateProvince: Buenos Aires

county: General Pueyrredón

municipality: Sierra de los Padres

locality: Paititi

verbatimCoordinates: 37°55'11.55"S 57°49'21.55"W?

verbatimSRS: WGS84

decimalLatitude: -36.080125

decimalLongitude: -56.177347222222

georeferenceProtocol: GPS

#### Identification

identifiedBy: Nelson Ferretti

dateIdentified: 2015

#### Geological context

#### Event

samplingProtocol: Pitfall trap

eventDate: 2015-06-16

year: 2015

month: 6

day: 16

#### Record Level

collectionCode: LZI

#### Material

Type status: Other material

##### Occurrence

catalogNumber: MACN6696

recordedBy: Cesari, Dominguez

individualCount: 1

sex: male

lifeStage: adult

##### Taxon

scientificNameID: Calathotarsus simoni

kingdom: Animalia

phylum: Arthropoda

class: Arachnida

order: Araneae

family: Migidae

genus: Calathotarsus

specificEpithet: simoni

scientificNameAuthorship: Schiapelli and Gerschman de Pikelín, 198

##### Location

continent: South America

country: Argentina

stateProvince: Buenos Aires

county: Tornquist

municipality: Tornquist

locality: Cerro Negro

##### Identification

##### Geological context

##### Event

eventDate: 1974-04

year: 1974

month: 4

##### Record Level

collectionCode: MACN

#### Occurrence

catalogNumber: MACN6696

recordedBy: Cesari, Dominguez

individualCount: 1

sex: male

lifeStage: adult

#### Taxon

scientificNameID: Calathotarsus simoni

kingdom: Animalia

phylum: Arthropoda

class: Arachnida

order: Araneae

family: Migidae

genus: Calathotarsus

specificEpithet: simoni

scientificNameAuthorship: Schiapelli and Gerschman de Pikelín, 198

#### Location

continent: South America

country: Argentina

stateProvince: Buenos Aires

county: Tornquist

municipality: Tornquist

locality: Cerro Negro

#### Identification

#### Geological context

#### Event

eventDate: 1974-04

year: 1974

month: 4

#### Record Level

collectionCode: MACN

#### Material

Type status: Other material

##### Occurrence

catalogNumber: MACN36114

recordedBy: Cesari

individualCount: 3

sex: 1 male, 2 females

lifeStage: adult

##### Taxon

scientificNameID: Calathotarsus simoni

kingdom: Animalia

phylum: Arthropoda

class: Arachnida

order: Araneae

family: Migidae

genus: Calathotarsus

specificEpithet: simoni

scientificNameAuthorship: Schiapelli and Gerschman de Pikelín, 199

##### Location

continent: South America

country: Argentina

stateProvince: Buenos Aires

county: Tornquist

municipality: Tornquist

locality: Cerro Negro

##### Identification

identifiedBy: Pablo Goloboff

##### Geological context

##### Event

eventDate: 1974-04

year: 1974

month: 4

##### Record Level

collectionCode: MACN

#### Occurrence

catalogNumber: MACN36114

recordedBy: Cesari

individualCount: 3

sex: 1 male, 2 females

lifeStage: adult

#### Taxon

scientificNameID: Calathotarsus simoni

kingdom: Animalia

phylum: Arthropoda

class: Arachnida

order: Araneae

family: Migidae

genus: Calathotarsus

specificEpithet: simoni

scientificNameAuthorship: Schiapelli and Gerschman de Pikelín, 199

#### Location

continent: South America

country: Argentina

stateProvince: Buenos Aires

county: Tornquist

municipality: Tornquist

locality: Cerro Negro

#### Identification

identifiedBy: Pablo Goloboff

#### Geological context

#### Event

eventDate: 1974-04

year: 1974

month: 4

#### Record Level

collectionCode: MACN

#### Material

Type status: Other material

##### Occurrence

catalogNumber: MACN36113

recordedBy: Cesari

individualCount: 1

sex: male

lifeStage: adult

##### Taxon

scientificNameID: Calathotarsus simoni

kingdom: Animalia

phylum: Arthropoda

class: Arachnida

order: Araneae

family: Migidae

genus: Calathotarsus

specificEpithet: simoni

scientificNameAuthorship: Schiapelli and Gerschman de Pikelín, 200

##### Location

continent: South America

country: Argentina

stateProvince: Buenos Aires

county: Tornquist

municipality: Tornquist

locality: Cerro Negro

##### Identification

##### Geological context

##### Event

eventDate: 1974-04

year: 1974

month: 4

##### Record Level

collectionCode: MACN

#### Occurrence

catalogNumber: MACN36113

recordedBy: Cesari

individualCount: 1

sex: male

lifeStage: adult

#### Taxon

scientificNameID: Calathotarsus simoni

kingdom: Animalia

phylum: Arthropoda

class: Arachnida

order: Araneae

family: Migidae

genus: Calathotarsus

specificEpithet: simoni

scientificNameAuthorship: Schiapelli and Gerschman de Pikelín, 200

#### Location

continent: South America

country: Argentina

stateProvince: Buenos Aires

county: Tornquist

municipality: Tornquist

locality: Cerro Negro

#### Identification

#### Geological context

#### Event

eventDate: 1974-04

year: 1974

month: 4

#### Record Level

collectionCode: MACN

#### Material

Type status: Other material

##### Occurrence

catalogNumber: MACN6698

recordedBy: Maury

individualCount: 2

sex: female

lifeStage: adult

##### Taxon

scientificNameID: Calathotarsus simoni

kingdom: Animalia

phylum: Arthropoda

class: Arachnida

order: Araneae

family: Migidae

genus: Calathotarsus

specificEpithet: simoni

scientificNameAuthorship: Schiapelli and Gerschman de Pikelín, 201

##### Location

continent: South America

country: Argentina

stateProvince: Buenos Aires

county: Tornquist

municipality: Tornquist

locality: Fortin Chaco

##### Identification

##### Geological context

##### Event

eventDate: 1973-03

year: 1973

month: 3

##### Record Level

collectionCode: MACN

#### Occurrence

catalogNumber: MACN6698

recordedBy: Maury

individualCount: 2

sex: female

lifeStage: adult

#### Taxon

scientificNameID: Calathotarsus simoni

kingdom: Animalia

phylum: Arthropoda

class: Arachnida

order: Araneae

family: Migidae

genus: Calathotarsus

specificEpithet: simoni

scientificNameAuthorship: Schiapelli and Gerschman de Pikelín, 201

#### Location

continent: South America

country: Argentina

stateProvince: Buenos Aires

county: Tornquist

municipality: Tornquist

locality: Fortin Chaco

#### Identification

#### Geological context

#### Event

eventDate: 1973-03

year: 1973

month: 3

#### Record Level

collectionCode: MACN

#### Material

Type status: Other material

##### Occurrence

catalogNumber: MACN6643

recordedBy: Maury, Cesari

individualCount: 1

sex: female

lifeStage: adult

##### Taxon

scientificNameID: Calathotarsus simoni

kingdom: Animalia

phylum: Arthropoda

class: Arachnida

order: Araneae

family: Migidae

genus: Calathotarsus

specificEpithet: simoni

scientificNameAuthorship: Schiapelli and Gerschman de Pikelín, 202

##### Location

continent: South America

country: Argentina

stateProvince: Buenos Aires

county: Tornquist

municipality: Tornquist

locality: Fortin Chaco

##### Identification

##### Geological context

##### Event

eventDate: 1974-04

year: 1974

month: 4

##### Record Level

collectionCode: MACN

#### Occurrence

catalogNumber: MACN6643

recordedBy: Maury, Cesari

individualCount: 1

sex: female

lifeStage: adult

#### Taxon

scientificNameID: Calathotarsus simoni

kingdom: Animalia

phylum: Arthropoda

class: Arachnida

order: Araneae

family: Migidae

genus: Calathotarsus

specificEpithet: simoni

scientificNameAuthorship: Schiapelli and Gerschman de Pikelín, 202

#### Location

continent: South America

country: Argentina

stateProvince: Buenos Aires

county: Tornquist

municipality: Tornquist

locality: Fortin Chaco

#### Identification

#### Geological context

#### Event

eventDate: 1974-04

year: 1974

month: 4

#### Record Level

collectionCode: MACN

#### Material

Type status: Other material

##### Occurrence

catalogNumber: MACN6617

recordedBy: Cesari

individualCount: 1

sex: female

lifeStage: adult

##### Taxon

scientificNameID: Calathotarsus simoni

kingdom: Animalia

phylum: Arthropoda

class: Arachnida

order: Araneae

family: Migidae

genus: Calathotarsus

specificEpithet: simoni

scientificNameAuthorship: Schiapelli and Gerschman de Pikelín, 203

##### Location

continent: South America

country: Argentina

stateProvince: Buenos Aires

county: Balcarce

municipality: Balcarce

locality: Sierra de la Cruz

##### Identification

##### Geological context

##### Event

eventDate: 1972-02

year: 1972

month: 2

##### Record Level

collectionCode: MACN

#### Occurrence

catalogNumber: MACN6617

recordedBy: Cesari

individualCount: 1

sex: female

lifeStage: adult

#### Taxon

scientificNameID: Calathotarsus simoni

kingdom: Animalia

phylum: Arthropoda

class: Arachnida

order: Araneae

family: Migidae

genus: Calathotarsus

specificEpithet: simoni

scientificNameAuthorship: Schiapelli and Gerschman de Pikelín, 203

#### Location

continent: South America

country: Argentina

stateProvince: Buenos Aires

county: Balcarce

municipality: Balcarce

locality: Sierra de la Cruz

#### Identification

#### Geological context

#### Event

eventDate: 1972-02

year: 1972

month: 2

#### Record Level

collectionCode: MACN

#### Material

Type status: Other material

##### Occurrence

catalogNumber: MACN6622

recordedBy: Maury, Cesari, Dominguez

individualCount: 1

sex: female

lifeStage: adult

##### Taxon

scientificNameID: Calathotarsus simoni

kingdom: Animalia

phylum: Arthropoda

class: Arachnida

order: Araneae

family: Migidae

genus: Calathotarsus

specificEpithet: simoni

scientificNameAuthorship: Schiapelli and Gerschman de Pikelín, 204

##### Location

continent: South America

country: Argentina

stateProvince: Buenos Aires

county: Tornquist

municipality: Tornquist

locality: Fortin Chaco

##### Identification

##### Geological context

##### Event

##### Record Level

collectionCode: MACN

#### Occurrence

catalogNumber: MACN6622

recordedBy: Maury, Cesari, Dominguez

individualCount: 1

sex: female

lifeStage: adult

#### Taxon

scientificNameID: Calathotarsus simoni

kingdom: Animalia

phylum: Arthropoda

class: Arachnida

order: Araneae

family: Migidae

genus: Calathotarsus

specificEpithet: simoni

scientificNameAuthorship: Schiapelli and Gerschman de Pikelín, 204

#### Location

continent: South America

country: Argentina

stateProvince: Buenos Aires

county: Tornquist

municipality: Tornquist

locality: Fortin Chaco

#### Identification

#### Geological context

#### Event

#### Record Level

collectionCode: MACN

#### Material

Type status: Other material

##### Occurrence

catalogNumber: MACN6697

recordedBy: Maury, Cesari, Dominguez

individualCount: 2

sex: female

lifeStage: adult

##### Taxon

scientificNameID: Calathotarsus simoni

kingdom: Animalia

phylum: Arthropoda

class: Arachnida

order: Araneae

family: Migidae

genus: Calathotarsus

specificEpithet: simoni

scientificNameAuthorship: Schiapelli and Gerschman de Pikelín, 205

##### Location

continent: South America

country: Argentina

stateProvince: Buenos Aires

county: Balcarce

municipality: Balcarce

locality: La Barrosa

##### Identification

##### Geological context

##### Event

eventDate: 1974-07

year: 1974

month: 7

##### Record Level

collectionCode: MACN

#### Occurrence

catalogNumber: MACN6697

recordedBy: Maury, Cesari, Dominguez

individualCount: 2

sex: female

lifeStage: adult

#### Taxon

scientificNameID: Calathotarsus simoni

kingdom: Animalia

phylum: Arthropoda

class: Arachnida

order: Araneae

family: Migidae

genus: Calathotarsus

specificEpithet: simoni

scientificNameAuthorship: Schiapelli and Gerschman de Pikelín, 205

#### Location

continent: South America

country: Argentina

stateProvince: Buenos Aires

county: Balcarce

municipality: Balcarce

locality: La Barrosa

#### Identification

#### Geological context

#### Event

eventDate: 1974-07

year: 1974

month: 7

#### Record Level

collectionCode: MACN

#### Material

Type status: Other material

##### Occurrence

catalogNumber: LZI543

recordedBy: Ferretti

individualCount: 1

sex: female

lifeStage: adult

##### Taxon

scientificNameID: Calathotarsus simoni

kingdom: Animalia

phylum: Arthropoda

class: Arachnida

order: Araneae

family: Migidae

genus: Calathotarsus

specificEpithet: simoni

scientificNameAuthorship: Schiapelli and Gerschman de Pikelín, 206

##### Location

continent: South America

country: Argentina

stateProvince: Buenos Aires

county: Balcarce

municipality: Balcarce

locality: La Barrosa

verbatimCoordinates: 37°52'37"S 58°15'40"W?

verbatimSRS: WGS84

decimalLatitude: -36.123055555556

decimalLongitude: -57.738888888889

georeferenceProtocol: GPS

##### Identification

identifiedBy: Ferretti

dateIdentified: 2015

##### Geological context

##### Event

samplingProtocol: Hand collected

eventDate: 2015-11-12

year: 2015

month: 11

day: 12

##### Record Level

collectionCode: LZI

#### Occurrence

catalogNumber: LZI543

recordedBy: Ferretti

individualCount: 1

sex: female

lifeStage: adult

#### Taxon

scientificNameID: Calathotarsus simoni

kingdom: Animalia

phylum: Arthropoda

class: Arachnida

order: Araneae

family: Migidae

genus: Calathotarsus

specificEpithet: simoni

scientificNameAuthorship: Schiapelli and Gerschman de Pikelín, 206

#### Location

continent: South America

country: Argentina

stateProvince: Buenos Aires

county: Balcarce

municipality: Balcarce

locality: La Barrosa

verbatimCoordinates: 37°52'37"S 58°15'40"W?

verbatimSRS: WGS84

decimalLatitude: -36.123055555556

decimalLongitude: -57.738888888889

georeferenceProtocol: GPS

#### Identification

identifiedBy: Ferretti

dateIdentified: 2015

#### Geological context

#### Event

samplingProtocol: Hand collected

eventDate: 2015-11-12

year: 2015

month: 11

day: 12

#### Record Level

collectionCode: LZI

#### Extent of occurrence

EOO (km2): 7207

Trend: Decline (inferred)

Justification for trend: This species only inhabits mountainous grassland in southern Buenos Aires province. Due to its microhabitat requirements, *C. simoni* is not able to occupy the plains and meadows between the two mountain systems where it is known from. Much of its range is currently being invaded by alien woody plants into relict native grasslands, comprising an important threat to its populations (Zalba and Villamil 2002). The range is also impacted by intensive cattle production (Pucheta et al. 1998) and overgrazing by feral horses (Zalba and Cozzani 2004), both of which negatively affect the species through microhabitat modification.

Causes ceased?: Unknown

Causes understood?: Unknown

Causes reversible?: Unknown

Extreme fluctuations?: Unknown

#### Area of occupancy

Trend: Decline (inferred)

Justification for trend: Due to specific habitat requirements, the populations are restricted to just some particular environments inside the species range. The species only survives in hilly and rocky grassland areas at about 500-1500 meters above sea level. The areas where *C. simoni* lives comprise steep shaded slopes with a moist substrate with particular moss species where the spiders are able to build their burrows (Schiapelli and Gerschman de Pikelín 1973, Ferretti et al. 2014a). A recent invasion of alien tree species (e.g. *Pinus halepensis*) can clearly promote changes in the substrate conditions and microenvironment (Zalba et al. 2008). In addition, no individuals were found at some of the original collection sites and from the Argentinean scientific collections revised, only 10 individuals were found after its description (all from a single site).

Causes ceased?: Unknown

Causes understood?: Unknown

Causes reversible?: Unknown

Extreme fluctuations?: Unknown

AOO (km2): 16

#### Locations

Number of locations: 2

Justification for number of locations: Two locations are identified due to the invasion of the species habitat by alien woody plants, exotic tree and shrub species with a consequent modification and loss of the native habitat. Given the proximity of some of the sites the invading species are covering multiple sites following single introduction events.

Trend: Stable

Justification for trend: No locations were lost during the last 10 years or three generations, so we can infer a stable number of locations.

Extreme fluctuations?: Unknown

#### Population

Trend: Decline (inferred)

Basis for decline: (c) a decline in area of occupancy, extent of occurrence and/or quality of habitat

Causes ceased?: Unknown

Causes understood?: Unknown

Causes reversible?: Unknown

Extreme fluctuations?: Unknown

**Figure 4.**
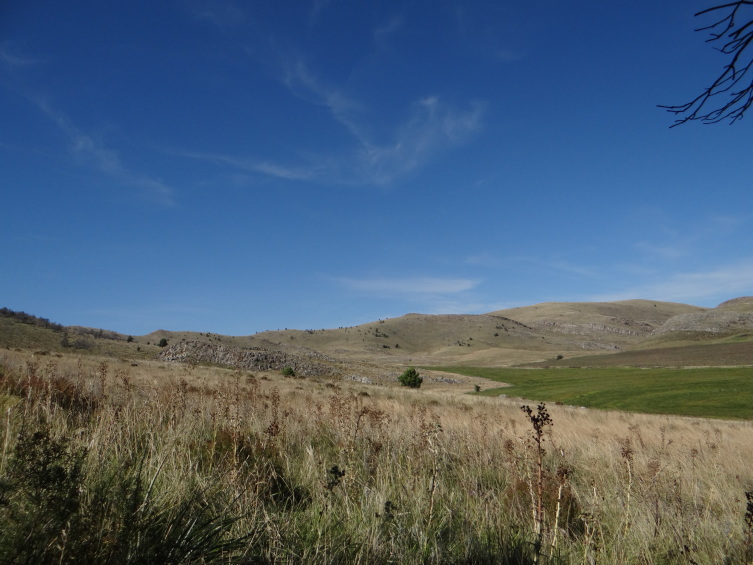
Habitat of *C. simoni* in Ventania

Population Information (Narrative): Four subpopulations are known for this species. Two subpopulations are located at the Ventania mountain system Fig. 4 and the other two subpopulations are located at the Tandilia mountain system.

#### Subpopulations

Number of subpopulations: 4

Trend: Decline (inferred)

Extreme fluctuations?: Unknown

Severe fragmentation?: Yes

Justification for fragmentation: Ferretti et al. (2014a) highlighted the rarity of this species in nature and the specific microclimatic conditions that it needs to survive. A decrease in AOO is related to a recent invasion of alien tree species (e.g. *Pinus halepensis*) that clearly promotes changes in the substrate conditions and microenvironment (Zalba and Villamil 2002). Despite the effort to locate some of the subpopulations reported on the original description of the species, for example inside the Ernesto Tornquist Provincial Park, no individuals were found after two consecutive years of surveys (Ferretti et al. 2012). Only about 50% of burrows studied by Ferretti et al. (2014a) were occupied by adult individuals, all of these being females. During subsequent field campaigns, only one adult male was seen and that corresponded to a juvenile specimen later molting in laboratory conditions. This could be due to the short life span of males, as for other trapdoor species they are short-lived wandering when adulthood is reached (Gupta et al. 2015).

#### Habitat

System: Terrestrial

Habitat specialist: Yes

**Figure 5.**
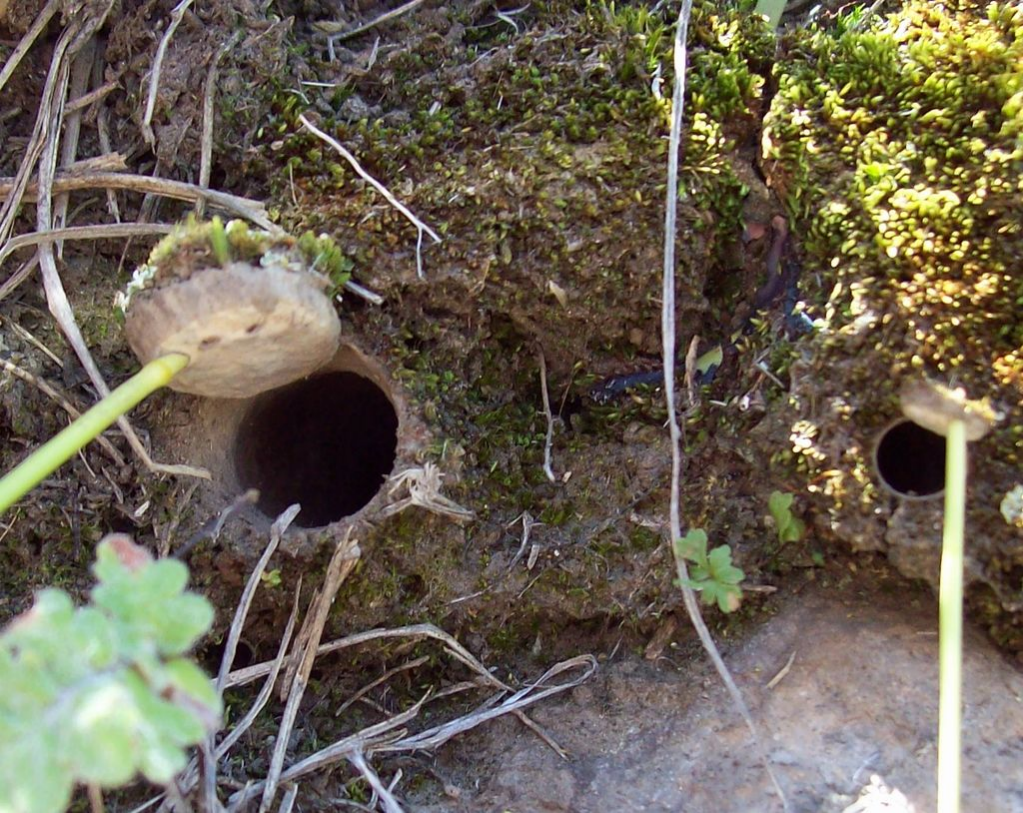
Aggregation of trapdoors of *C. simoni* in nature

Habitat (narrative): This species is very rare on nature and very difficult to find due to the specific microclimatic conditions that it needs. It only inhabits steep shaded and very humid slopes, with burrows only found in association to some particular species of mosses Fig. 5.

Trend in extent, area or quality?: Decline (estimated)

Justification for trend: Habitat loss, fragmentation and modification comprise the major threats and could cause local extirpations in the near future. The mountain systems of Ventania and Tandilia show the highest intensities across Argentina of woody species invasions. These regions were totally treeless at the end of the nineteenth century (Spegazzini 1896) corresponding to the native grassland habitat, but at the present time they are populated by numerous exotic tree and shrub species. Pines were originally planted in these areas during the 1950s, with trees clustered in small plantations and also spread along trails and roadsides. Currently, the locations of *C. simoni* are undergoing severe invasion by those trees that deeply transform the structure and composition of native grasslands and can also enhance the invasion by other exotic species (Zalba et al. 2008).

##### Habitat

Habitat importance: Major Importance

Habitats: 4. Grassland6. Rocky areas (e.g. inland cliffs, mountain peaks)

#### Habitat

Habitat importance: Major Importance

Habitats: 4. Grassland6. Rocky areas (e.g. inland cliffs, mountain peaks)

#### Ecology

Size: 12-20 mm

Generation length (yr): 3

Dependency of single sp?: Unknown

**Figure 6.**
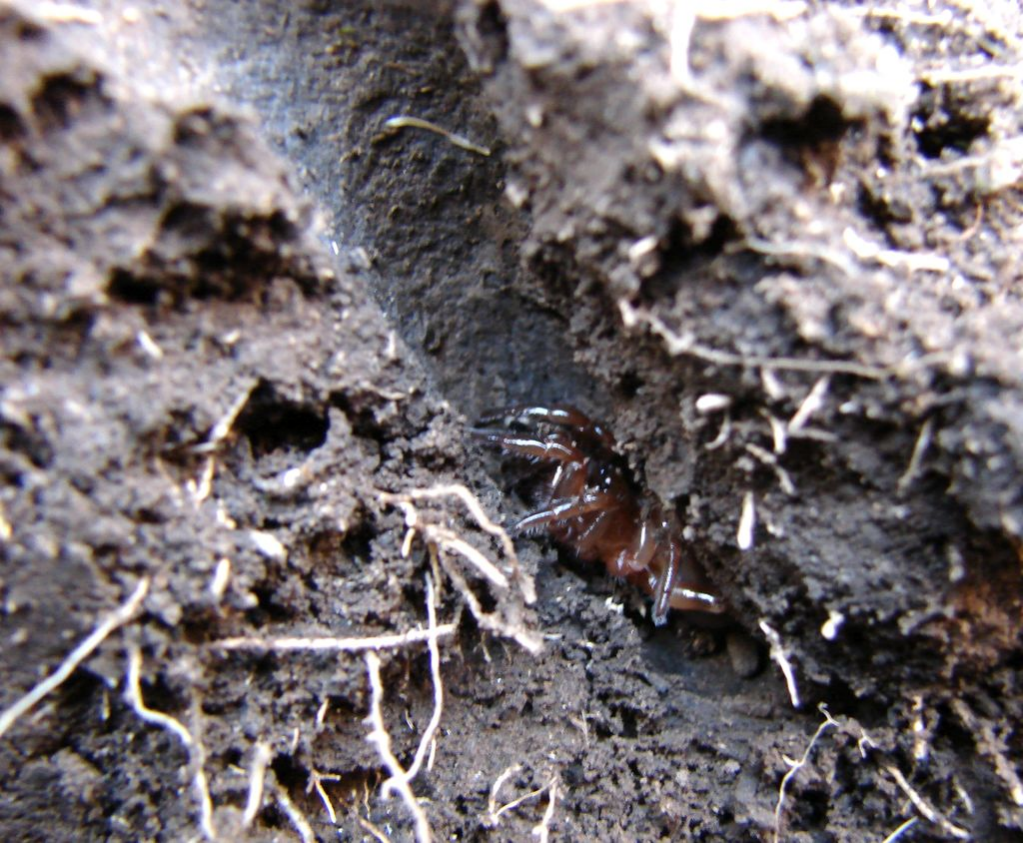
Burrow shape of a juvenile of *C. simoni*

Ecology and traits (narrative): The mean density of adults recorded on a steep slope was 0.01/m2 and mean density of juveniles was 0.009/m2 (Ferretti et al. 2014a). The trapdoor is relatively thick and rigid with beveled edges and, when closed, its edges fit snugly into the tough entrance rim, which flares outward to form a complementary bevel. The door is connected to the entrance rim by a narrow but firmly articulated hinge. The entrance rim is usually nearly flush with the surrounding soil. Its inner surface is covered with a thick, tough white layer of silk, and its outer surface, which is made of soil with abundant mosses and lichens, resembles the surrounding ground surface. Most of the burrows extended roughly straight back into the soil accumulated between rocks of a hillside, approximately perpendicular to the surface Fig. 6. Data on reproduction biology are scarce. Females build spherical egg sacs, containing approximately 20 eggs. Juveniles emerge during November and December, the spring and summer seasons. From the work carried out by (Ferretti et al. 2014a) in a study area of about 2350 m^2^, a number of 30 adult individual (just females) was reported, based on active searching for burrows and using pitfall traps. Adult males of this species are extremely hard to find and nothing is known about the courtship and mating behavior of this species.

#### Threats

Justification for threats: See “Extent of occurrence”

##### Threats

Threat type: Ongoing

Threats: 2. Agriculture & aquaculture2.1. Agriculture & aquaculture - Annual & perennial non-timber crops2.3. Agriculture & aquaculture - Livestock farming & ranching8. Invasive and other problematic species, genes & diseases8.1. Invasive and other problematic species, genes & diseases - Invasive non-native/alien species/diseases

#### Threats

Threat type: Ongoing

Threats: 2. Agriculture & aquaculture2.1. Agriculture & aquaculture - Annual & perennial non-timber crops2.3. Agriculture & aquaculture - Livestock farming & ranching8. Invasive and other problematic species, genes & diseases8.1. Invasive and other problematic species, genes & diseases - Invasive non-native/alien species/diseases

#### Conservation

Justification for conservation actions: This species occurs near the “Ernesto Tornquist” Provincial Park in Ventania. However, no specimens were found inside this natural reserve (Ferretti et al. 2012). In Tandilia mountain system, one location is protected under a recently created private natural reserve. The area management inside these natural reserves involving reduction of alien species invasion (such as pines) could possibly increase the area if adequate habitat (Cuevas and Zalba 2010). Also, the installation of informative panels educating the visitors and public talks to park rangers about this rare endemic species could increase the awareness of the mountain grassland as a natural habitat that deserves protection.

##### Conservation actions

Conservation action type: In Place

Conservation actions: 1. Land/water protection1.1. Land/water protection - Site/area protection1.2. Land/water protection - Resource & habitat protection2. Land/water management2.1. Land/water management - Site/area management

##### Conservation actions

Conservation action type: Needed

Conservation actions: 4. Education & awareness

#### Conservation actions

Conservation action type: In Place

Conservation actions: 1. Land/water protection1.1. Land/water protection - Site/area protection1.2. Land/water protection - Resource & habitat protection2. Land/water management2.1. Land/water management - Site/area management

#### Conservation actions

Conservation action type: Needed

Conservation actions: 4. Education & awareness

#### Other

##### Use and trade

Use type: International

##### Ecosystem services

#### Use and trade

Use type: International

#### Ecosystem services

#### Viability analysis

## Supplementary Material

Supplementary material 1 Range Map Data type: Kml Google Map
File: oo_133016.kml Cardoso, P

## Figures and Tables

**Figure 2.**
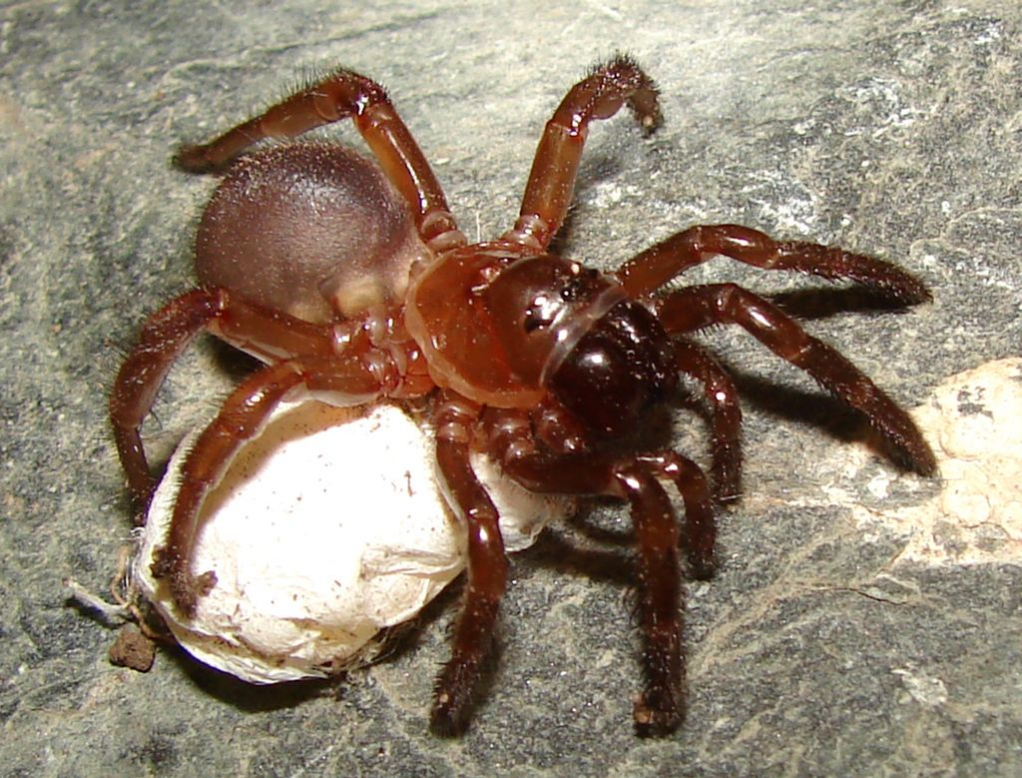
Adult female of *C. simoni* holding an egg sac
